# P40 Evaluation of sulbactam/durlobactam activity against carbapenem-resistant *Acinetobacter baumannii* in India: an institutional experience

**DOI:** 10.1093/jacamr/dlaf230.047

**Published:** 2025-12-04

**Authors:** M Nizam Ahmed, Parul Singh, Madhavi Kirti, Bharat Chandra Das, Vanlal Tluanpuii, Subodh Kumar, Sushma Sagar, Kapil Dev Soni, Richa Aggarwal, Ashish Bindra, Keshav Goyal, Gyanendra Pal Singh, Navdeep Sokhal, Kamran Farooque, Purva Mathur

**Affiliations:** All India Institute of Medical Sciences, New Delhi, India; All India Institute of Medical Sciences, New Delhi, India; All India Institute of Medical Sciences, New Delhi, India; All India Institute of Medical Sciences, New Delhi, India; All India Institute of Medical Sciences, New Delhi, India; All India Institute of Medical Sciences, New Delhi, India; All India Institute of Medical Sciences, New Delhi, India; Trauma Centre, All India Institute of Medical Sciences, New Delhi, India; Trauma Centre, All India Institute of Medical Sciences, New Delhi, India; Trauma Centre, All India Institute of Medical Sciences, New Delhi, India; Trauma Centre, All India Institute of Medical Sciences, New Delhi, India; Trauma Centre, All India Institute of Medical Sciences, New Delhi, India; Trauma Centre, All India Institute of Medical Sciences, New Delhi, India; Trauma Centre, All India Institute of Medical Sciences, New Delhi, India; All India Institute of Medical Sciences, New Delhi, India

## Abstract

**Background:**

Carbapenem-resistant *Acinetobacter baumannii* (CRAB) is a WHO designated ‘critical priority’ pathogen because of its high capacity for multidrug resistance and association with mortality rates approaching 50% in severe hospital-acquired infections. CRAB is a major driver of ventilator-associated pneumonia (VAP), bloodstream infections and wound infections in critically ill patients, especially in intensive care units. Treatment options are severely limited, with colistin and minocycline often used as last-resort agents despite toxicity concerns. The recently concluded ATTACK trial demonstrated that sulbactam/durlobactam, a novel β-lactam/β-lactamase inhibitor combination, was superior to colistin in terms of efficacy and safety, establishing it as a preferred therapeutic option in global guidelines. Surveillance programmes have consistently reported very high activity, with more than 96% of CRAB isolates worldwide remaining susceptible. However, antimicrobial resistance (AMR) in India is distinct, with carbapenem resistance driven not only by OXA-type carbapenemases but also by endemic metallo-β-lactamases (MBLs) and frequent treatment exposures to broad-spectrum agents. Therefore, regional evaluation of sulbactam/durlobactam is crucial before incorporation into local treatment algorithms.

**Methods:**

A prospective laboratory-based surveillance was conducted over four months at a tertiary-care teaching hospital. A total of 175 non-duplicate CRAB clinical isolates were collected. The majority originated from respiratory samples (*n*=140; 80%), reflecting the heavy burden of ventilator-associated and hospital-acquired pneumonia. Additional sources included blood (*n*=18; 10%), pus/wound swabs (*n*=7; 4%) and other sterile body fluids (*n*=10; 6%). All isolates were confirmed as *A. baumannii* by VITEK MS^®^ (bioMérieux^®^, France). Antimicrobial susceptibility testing was performed by disc diffusion, and sulbactam/durlobactam activity was assessed using CLSI-M100 2024. *A. baumannii* SAMN04901667(AR Bank) was included as a quality control strain. Comparative susceptibility to colistin and minocycline was also assessed.

**Results:**

Sulbactam/durlobactam exhibited susceptibility in 120 of 175 isolates (68.6%). Non-susceptibility was noted in 31.4% of isolates, substantially higher than resistance rates reported in global surveillance (<2%) or European studies (87.9–96% susceptibility). In contrast, minocycline and colistin demonstrated higher retention of activity, with susceptibility rates of 92.0% (161/175) and 94.0% (164/175), respectively. Respiratory isolates dominated the dataset, accounting for 80% of cases, consistent with India’s disproportionate burden of ventilator-associated pneumonia. No difference in sulbactam/durlobactam susceptibility was observed across specimen types.

**Conclusions:**

This study provides the first institutional-level data from India on sulbactam/durlobactam activity against CRAB. Unlike international reports where the agent has emerged as a highly reliable option, our findings reveal considerably reduced susceptibility, with nearly one-third of isolates resistant. This discrepancy likely reflects region-specific resistance mechanisms, such as mutations in penicillin-binding protein 3 (PBP3), the target of sulbactam, or the presence of MBLs that confer cross-resistance. The high prevalence of combination resistance mechanisms in Indian CRAB strains may further compromise efficacy.

